# Rapidly Self-Sterilizing PPE Capable of Destroying 100% of Microbes in 30-60 Seconds

**DOI:** 10.3389/fcimb.2021.752899

**Published:** 2021-12-15

**Authors:** Alfred A. Zinn, Mina Izadjoo, Hosan Kim, Rachel L. Brody, Robert R. Roth, Agustin Vega, Khanh K. Nguyen, Nhi T. Ngo, Hannah T. Zinn, Nicholas Antonopoulos, Randall M. Stoltenberg

**Affiliations:** ^1^ Kuprion^®^, Inc., San Jose, CA, United States; ^2^ Integrated Pharma Services, Frederick, MD, United States

**Keywords:** copper, antimicrobial, antibiotic resistant bacteria, ultra-active, antiviral, novel material

## Abstract

The continued proliferation of superbugs in hospitals and the coronavirus disease 2019 (COVID-19) has created an acute worldwide demand for sustained broadband pathogen suppression in households, hospitals, and public spaces. In response, we have created a highly active, self-sterilizing copper configuration capable of inactivating a wide range of bacteria and viruses in 30-60 seconds. The highly active material destroys pathogens faster than any conventional copper configuration and acts as quickly as alcohol wipes and hand sanitizers. Unlike the latter, our copper material does not release volatile compounds or leave harmful chemical residues and maintains its antimicrobial efficacy over sustained use; it is shelf stable for years. We have performed rigorous testing in accordance with guidelines from U.S. regulatory agencies and believe that the material could offer broad spectrum, non-selective defense against most microbes *via* integration into masks, protective equipment, and various forms of surface coatings.

## Highlights

A novel configuration of copper offering continued fast-acting protection against a wide range of viruses and bacteria.

## Introduction

The rush on PPE and bare supply stockrooms that followed during the pandemic’s onset illustrated a fatal flaw in our approach to microbe management. The world relies on stockpiles of resources to create a clean field that is sullied immediately upon contact. The sterility provided by widely used disinfectant options is all too fleeting. Demands imposed by COVID-19 turned inefficiencies into breaking points; there is simply not enough sterile equipment in the world to shield ourselves if materials do not offer protection beyond a single use. The masks we are using accumulate bacteria over the course of normal wear and are thus limited to a single, short use before they must be washed or discarded (Yang et al., 2018). We need a material that keeps hands, surfaces, and tools free of pathogens over periods of time spanning weeks, months, or years.

This glaring void in currently available antimicrobial products and the impending threat of antibiotic resistance led us to hone our engineered copper material (ECM) from a lead-free solder alternative into an ultra-active antimicrobial. Catalysis taught us that a material’s surface reactivity is contingent on its surface area and energy; the greater the surface area, the more reactive it will be (Nilsson et al., 2008; Hammond and Conner, 2013). Our ECM owes its activity to a large interconnected meso-structured metal network consisting of a polymeric gel-like copper phase with meso-scale porosity and surface roughness. As COVID-19 overwhelmed the world in early 2020, we hypothesized that this unique structure and its surfactant shell should be highly antimicrobial. The literature is rife with examples of copper as an antimicrobial and the EPA has approved hundreds of copper alloys for their disinfectant properties (Grass et al., 2011; United States Environmental Protection Agency, 2018). However, they are slow-acting and require up to 4 hours to fully deactivate microbes. Because conventional copper surfaces disinfect more slowly, they in turn still need to be disinfected regularly. Given the structural differences between ECM and previous copper iterations, it made sense to explore ECM’s anti-microbial activity.

**Figure 1:**
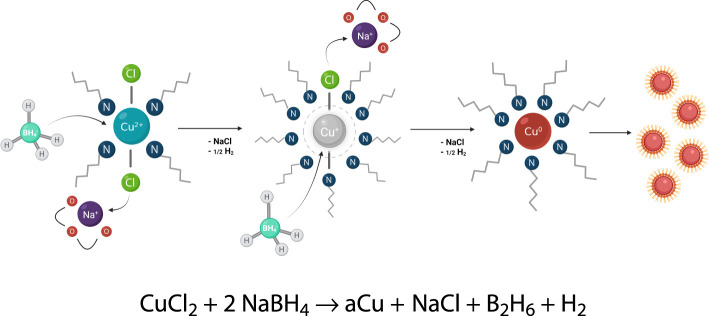
Synthesis of ECM *via* reduction of CuCl_2_. Anhydrous copper(II) chloride is reduced with sodium borohydride in the presence of amine surfactants to form the raw ECM material. The reaction takes place near room temperature. The borane generated during the reduction forms borane-amine complexes which are later hydrolyzed upon the addition of water to remove the NaCl byproduct. The amines assist in dissolving the copper(II) chloride and mediate resultant particle sizes. This prevents the ECM particles from oxidizing and continuing to grow.

**Figure 2:**
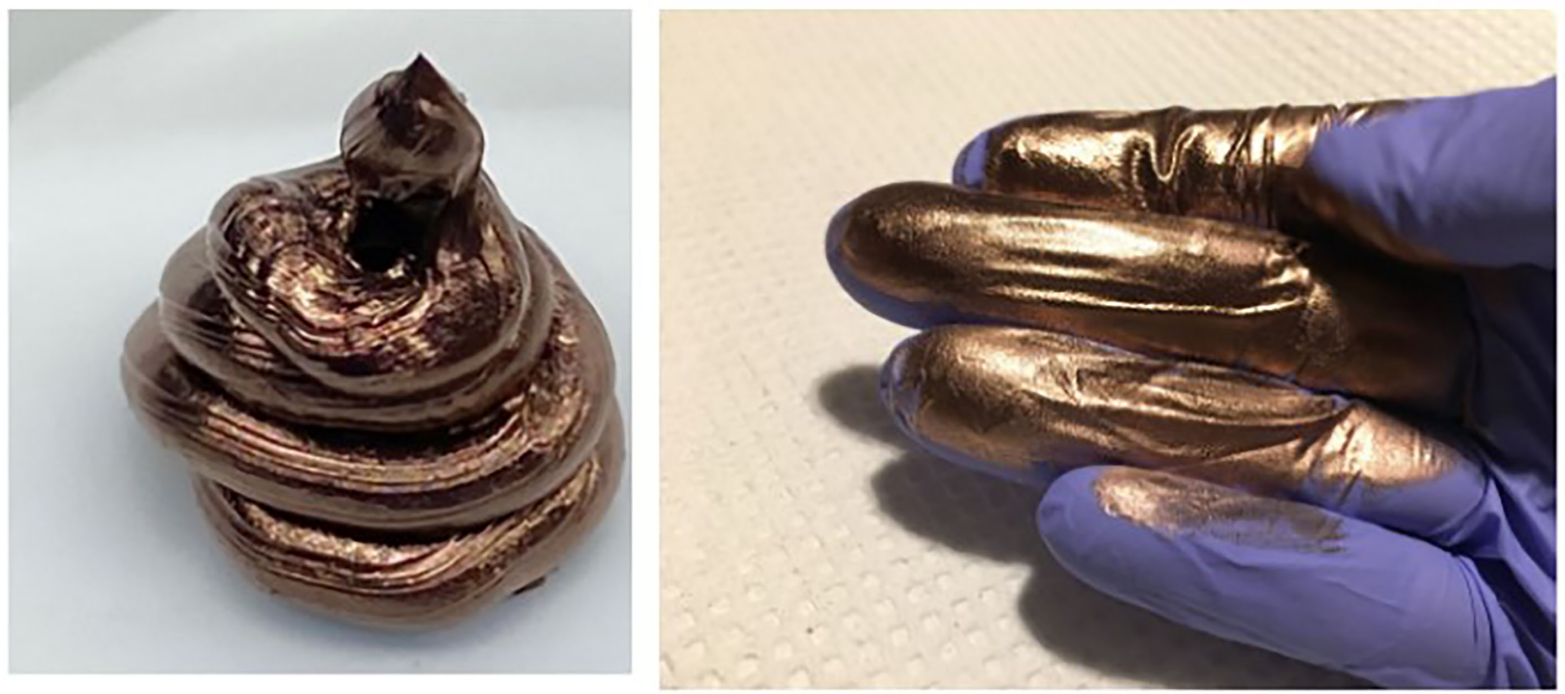
Images of ECM after being dispensed from a tube. The dense thixotropic copper gel is paste-like and stable. It behaves like a typical non-Newtonian “liquid”. A layer of amine surfactants lends oxidation protection and exerts strong cohesion *via* hydrophobic interactions and organic end-chain entanglement. It can be spread very thin with a lotion-like texture.

ECM is manufactured using a bottom-up synthesis approach *via* NaBH_4_ reduction of CuCl_2_ in solution (**Figure 1**). The resultant copper material is rendered stable through the formation of a very sticky paste-like metallic gel. It behaves like a typical non-Newtonian “liquid” with high thixotropy (**Figure 2**). The specially designed amine surfactant layer protects the copper from oxidation and exerts strong cohesion *via* hydrophobic interactions and organic end-chain entanglement, which holds the dense metallic gel together with the aid of the high surface area and energy and does not dissolve in water and most organic solvents. Its structure can be described as a dense but flowable metallic gel-like paste with an interconnected meso-scale network, porosity and surface roughness that enables safe handling and processing; it never turns into an airborne powder but rather hardens into a solid copper mass similar to a xerogel, allowing the material to retain the high activity of the unfused ECM.

**Figure 3:**
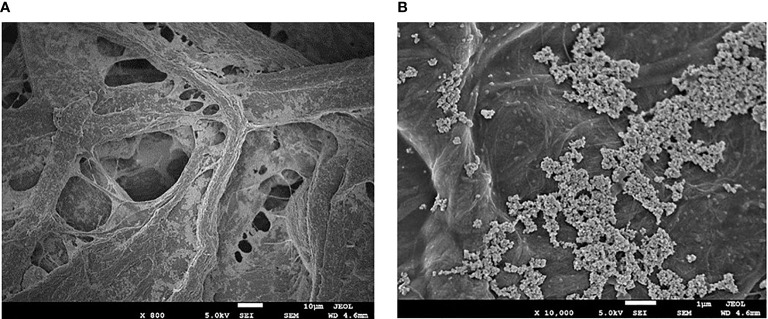
High magnification scanning electron microscopy images of ECM on 45/55 cellulose/polyester fabric. **(A)** fibers of the cellulose/polyester fabric coated with ECM. **(B)** 10,000x magnification of ECM on fabric, showing copper agglomerates in the 3-7 micrometer range.

We set out to quantify ECM’s activity against a host of viruses and bacteria with the eventual goal of testing against the SARS-CoV-2 virus. We formulated water-based paints to coat fabrics and porous filter materials that could be used in face masks and air purification units such as HVAC systems in airplanes, hospitals or public transportation (**Figure 3**). For clarity, we will refer to the antimicrobial applications of ECM as ActiveCopper (aCu). The Environmental Protection Agency (EPA) outlines a set of tests to measure the efficacy, longevity, and durability of copper alloys as antimicrobials. In each instance our antimicrobial tests followed the EPA protocols. We made the decision to modify test windows to increase the demand placed on our material. In practice, this meant shortening the pathogen-aCu contact time to precisely determine how quickly the antimicrobial action took place and lengthening the duration of multi-day, multi-inoculation tests to gauge how long aCu could be expected to remain active.

## Materials and Methods

### Synthesis of ECM

A 3% solution of CuCl_2_ (3 L of 0.5M) in dimethyldiglycol that contained a proprietary amine surfactant mixture was heated to 40°C under a nitrogen atmosphere and 1.4 L of a 3 M NaBH_4_ solution (1.4 M equivalent) in dimethyltriglycol added as the reducing agent. The reaction mixture was continuously stirred for another 20 min until a dark red-brown slurry has formed and then cooled to room temperature. The solids were isolated by centrifugation (2200 rpm for 7 min) and then washed with 7 L of water to remove (dissolve) the NaCl that formed during the reduction step and centrifuged a second time (2200 rpm for 7 min) to isolate the ECM. The reaction yields about 100 g of product (Zinn et al., 2016).

### Coating Formulation

The ActiveCopper material is washed with isopropanol and pentane and ultrasonically agitated using a Branson Ultra Sonifier model #550 at 70% power for 25 sec each followed by centrifugation using a Beckman Coulter Allegra X-14R Centrifuge at 870 RCF for 6 min. The washed ActiveCopper is added to isopropanol at 1.16% concentration by weight. This solution is again sonicated and then homogenized using Omni International homogenizer model #GLH850 at 10,000 rpm for 5 min resulting in a uniform dispersion that is then used to coat the various types of fabric.

### Coating Application

This solution is sprayed onto the fabric using a Paasche 62 sprayer (Paasche Airbrush Company, Kenosha, WI) at 20psi. After each coat, the fabric is dried with a heat gun. A total of 5 coats were applied with 3 coats on the front side and 2 coats on the backside.

### Fabric Analysis

The copper load is determined using an established quantitative photometric approach. A 22 mm disc is cut out and added to 1000 µL of a 7% nitric solution. This solution is heated to dissolve all copper present on the fabric sample. The solution is then diluted with 3000 µL of a buffer solution made up of 30% w/vol of citric acid and 38% vol/vol of a 25% ammonia solution in H_2_O, additional 100 µL of 25% ammonia water is added and 900 µL H_2_O is added. The total solution is filtered into a clean vial using a 0.22 µm PTFE membrane syringe filter (NEST Scientific, Rahway, NJ). The filtered solution is further diluted into a clean vial by 25 µL of filtered solution to 975 µL buffer solution and 3500 µL H_2_O. Lastly, 500 µL of a 0.5% w/vol of Bis(cyclohexanone) oxaldihydrazone (Cuprizon) in a 50% vol/vol of an ethanol-H_2_O solution is added as the colorimetric indicator. The cuprizone forms a blue complex with Cu^2+^ ions and the concentration corresponds directly to the extinction coefficient measured. A C-M5 Spectrophotometer (Konica Minolta, Ramsey, NJ) is used to determine % transmission and compared to the calibration curve established using four copper standards with different concentrations (2.88 µg, 2.4 µg, 1.6 µg, 0.96 µg per mL). The equation of the curve of the known standard is used to calculate the mg/in^2^ of ActiveCopper for a given sample.

### Antimicrobial Testing

#### Modified Antiviral Test Against Salmonella Enterica-Specific Bacteriophage Ø32

The following five tests were conducted by Dr. Mina Izadjoo and Dr. Hosan Kim at Integrated Pharma Services in Frederick, MD. Four aCu-coated samples (CS200323-1, CS200323-2, CS200325-8, CS200325-9) and one control (BS200323-1) (A(A), A(B), E(A), E(B), Cont.) were used in this initial screen of activity against *Salmonella enterica*-specific bacteriophage Ø32. The samples were plated and administered a 5x10^4^ PFU challenge of bacteriophage Ø32. After the predetermined incubation period of 5, 30, or 60 min, 0.2 ml Dey-Engley neutralizing broth (Becton, Dickinson and Company, Franklin Lakes, NJ) was applied to wash the bacteriophages. The samples were removed as quickly as possible to avoid additional contact time. The viral suspension was serially diluted with phosphate buffered saline (PBS, Sigma Aldrich, St. Louis, MO). 0.2 ml of the resultant phage solution was administered to individual tryptic soy agar (TSA, MP Biomedicals, LLC, Solon, OH) plates along with 0.2 ml of the overnight NR-170 culture. 5 ml of 0.7% top agar was added and swirled to adequately mix. TSA plates were incubated at 37°C for 18 hours before being examined for plaques. 100% virus elimination would be indicated by no plaques. This same procedure was repeated with aCu on rayon/polyester (70/30) fabric samples (BS200401-1 Control, CS200401-1, -2 Active).

#### Modified (Lengthened) EPA Long-Term Test

Twenty cellulose/polyester (45/55) fabric samples cut from batch CS200416-3 (active) and BS200416-1 (control) were used in this trial. Ten were untreated and used as controls, ten were coated with 3 front and 2 back applications of aCu. Samples were challenged with a 2.7 x 10^7^ PFU/ml titer of *Salmonella enterica*-specific bacteriophage Ø32. The samples were tested sequentially; all samples received inoculation on T1, 4/21/20, followed by a 24-hour incubation period. Sample 1 received a single inoculation (4/21), sample 2 received two inoculations (4/21, 4/22), sample 3 received three inoculations (4/21, 4/22, 4/23) and so on up to sample 10, which received ten inoculations (4/21-5/4) over the 14-day period. Inoculations were not administered on Sat/Sun (4/25, 4/26 & 5/2, 5/3). Once the incubation period had elapsed (either 24 hours on weekdays or 72 hours on weekends) the virus was recovered with 1 ml of Dey-Engley neutralizing broth followed by vortexing and centrifugation. The resultant solution was then mixed with *Salmonella enterica* NR-170 culture and 0.7% top agar poured onto a TSA plate. After an 18-hour incubation period, plates were examined for presence of viral plaques. The complete absence of plaques indicated that aCu had achieved a 100% kill rate. The control fabrics were administered the same protocol and displayed prolific plaques at each time point, indicating otherwise hospitable conditions for viral growth.

#### Modified (Shortened) Ultra-Rapid EPA Efficacy Test

Cellulose/polyester (45/55) fabric samples from batches BS200416-2, CS200416-3, and CS211022-2 coated with aCu were used in this short-term exposure test. Samples from BS200416-1 were used as a blank control. aCu coated fabric samples were placed on empty petri dishes and spotted with 0.2 ml of the *Salmonella enterica*-specific bacteriophage Ø32 titer in the center of the fabric. This application delivered 2.7 x 10^7^ PFU/ml of bacteriophage to the sample. After inoculation the samples were incubated at room temperature for T0, T30s, T1, T2, T2.5, T3, and T5 minutes. After the designated time had elapsed, 2 ml of Dey-Engley neutralizing solution was added to wash the bacteriophages. The samples were removed as quickly as possible to avoid additional contact time. The viral suspension was diluted with phosphate buffered saline (PBS). 0.2 ml of the resultant phage solution was administered to respective tryptic soy agar (TSA) plates along with 0.2 ml of the overnight NR-170 culture. 5 ml of 0.7% top agar was added and swirled to adequately mix. TSA plates were incubated at 37°C for 18 hours before being examined for plaques. 100% virus deactivation would be indicated by no plaques, however further growth of host bacteria would continue.

#### Modified (Lengthened) EPA Longevity Test

A 2x2 cm, 3-year old 3 mm thick piece of aCu-coated aluminum was placed on an empty plate. 100 µL of *Salmonella enterica* specific bacteriophage Ø32 phage titer (~3 x 10^4^ PFU/ml) was spotted on the center of the aCu-aluminum sample. The sample was incubated at room temperature (37°C) for the designated time periods (T0, T15 s, T30 s, T1 m, T1.5 m, T2 m, T3 m) before the addition of 1 ml of Dey-Engley neutralizing broth. The aluminum was promptly washed by wiping with 70% isopropyl alcohol (IPA, Decon Labs, Inc, King of Prussia, PA). The viral suspension was diluted with PBS. 0.1 ml of the resultant solution was spotted on a TSA plate with the addition of 0.1 ml of *Salmonella enterica* NR-170 overnight culture. 5 ml of top agar (0.7%) was added and swirled to mix adequately. The plates were incubated at 37°C overnight before undergoing examination for viral plaques. Absence of plaques indicates 100% kill of bacteriophage

#### Determination of Antiviral Activity of Textile Products Based on International Standard Organization (ISO) Test Method 18184:2019

All test specimens were prepared in sterilized, capped vials. 2x2 cm pieces of cellulose/polyester (45/55) fabric were used. Untreated control specimens came from batch BS200427-1, aCu treated active specimens came from batch CS200427-1 Samples were sterilized *via* ultraviolet radiation overnight and stored in sterilized vials prior to testing. 0.2 ml of the respective virus suspensions were deposited onto each specimen and caps were tightly closed. The test samples were incubated for 2 hours at room temperature. The blank control samples were immediately treated (T0) with 20 ml of wash solution, SCDLP (Casein Peptone Lecithin Polysorbate Broth with Tween 80) medium. The aCu-coated specimens were treated with 20 ml SCDLP medium after a 2-hour contact time. The vials were closed and agitated by vortexing for 5 seconds repeated 5 times to wash out the virus. Results from antiviral tests of treated textile were compared to results of control (untreated textile).

The wash out suspension from both the control and treated samples and the virus control suspension was serially diluted in Essential Minimum Essential Medium with 1% Penicillin/Streptomycin antibiotics. The Human Influenza A H1N1, Human Influenza A H3N2 and Calicivirus VR-782 viral suspensions were diluted with PBS to concentrations of 10^7^ pfu/ml from the original titer.

For antiviral analysis, host cells to be used for the plaque titer determination assay were prepared in tissue culture 6-well plates and incubated at 37 ˚C with 5% CO_2_ for up to 2 days. Host cells for the TCID_50_ cytopathic effect titer determination assay prepared in tissue culture 96-well plates and incubated at 37°C with 5% CO_2_ for 7 days. Each virus has a specific host cell: MDCK Cell ATCC CL-34 is used for H1N1 and H3N2 and CRFK Cell ATCC CL-94 is used for Calicivirus.

1. For TCID_50_ Determination – Behrens and Karber Method was used
  1. Y=X x10^a^
  2. a = ∑p – 0.5

### Safety Analysis

#### Copper Shedding/Breathing Simulation Test

Samples treated with various coats of aCu (BS200408-1 control, CS200401-2, -3, -4, -5, -6 active, **S1 Table**) were individually loaded into a 25 mm stainless steel filter holder (Millipore Sigma, Burlington, MA). Purified dry N_2_ was blown through the filter holder for 15 min at a rate of 4.1 slpm based on estimates of the peak air flow rate experienced by a typical respirator mask during normal breathing. Nitrogen was administered in both the “forward” and “reverse” directions to simulate inhalation/exhalation, and collect shedding activity data from both sides. Optical particle counting using bin sizes of 0.2, 0.3, 0.5, and 1.0 µm was conducted using an Apex R02 Particle Counter (Lighthouse, Fremont, CA) on the N_2_ after passing through the aCu-treated fabric. Condensation particle counting was conducted between an inclusive range of 8 nm-3 µm. A water bubbler trap was used to collect any shed materials from the exhausted N_2_. Inductively coupled plasma mass spectrometry (ICP-MS) was conducted to analyze the water trapping solutions from the flow-through. Filtered N_2_ was captured by a 47 mm filter trap (Millipore Sigma, Burlington, MA) and 0.1 µm Track-Etch filters. These filters were used to look for the evidence of shed metal using SEM-EDS. Samples were submitted to Millipore Imaging Lab in Bedford, MA for imaging.

#### Gas Chromatography-Mass Spectrometry Testing

Samples coated with aCu (CS200726-1, -2, -3, -4) and a blank control (BS200726-1) were sent to Torrent Labs (Milpitas, CA) for EPA standards SW 8260B and 8270C, volatile and semi-volatile organic compounds testing *via* GCMS. The fabric samples were tested after 8 hours of tumbling in deionized water or after 1 hour of agitation in acidic deionized water, depending on the test iteration. Per the method supplied by the EPA, *via* purge and trap the analytes are loaded into the capillary column of the gas chromatograph and flash evaporated to a second capillary for the analysis to take place. From the capillary column, analytes are transferred to the mass spectrometer. The mass spectra of the analytes are compared with analytic standards to determine the presence or absence of a particular VOC. A five-point calibration curve is used to quantify the results against the recorded standards.

#### Copper Oxidation State Analysis

Using X-ray power diffraction (XRD) (SmartLab SE, Rigaku, Akishima, Japan), our ActiveCopper material was analyzed to assess the oxidation state and determine if any oxidation was occurring by using the standard reference peaks of copper. Analysis was conducted with SmartLab Studio II software from Rigaku.

#### Statistical Analysis

Analysis of the results of tests conducted in triplicate was run in R Studio (PBC, Boston, MA) using the R Stats package (R Core Team, Foundation for Statistical Computing, Vienna, Austria). Shapiro-Wilks Normality test was conducted to assess distribution normality, followed by ANOVA with Tukey’s multiple comparisons of means test with a 95% confidence interval. Graphic visualizations were created with ggplot2 (ggplot2, Hadley Wickham).

## Results

**Figure 4:**
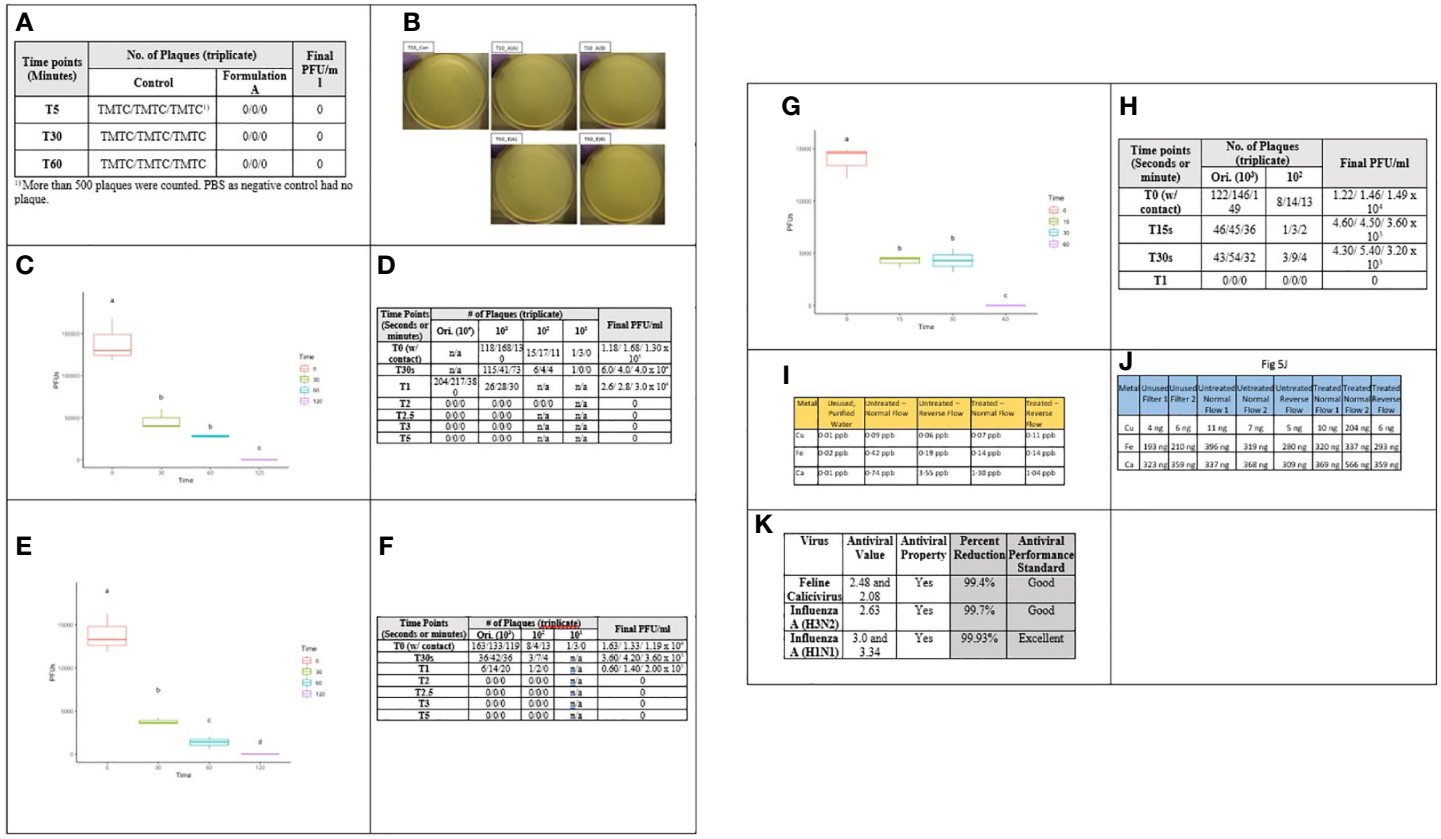
Results from antimicrobial tests of aCu. From left to right, **(A)** viral kill rate after 5, 30, and 60 minutes, compared to inactive control (4 active samples and 1 control per duration). **(B)** 100% kill rate of bacteriophage after 30 min on aCu-treated rayon/polyester fabric. **(C, D)** Deactivation of 3.0 x 10^5^ PFU/ml viral titer by formulation A of aCu fabric. **(E, F)** Deactivation of 3.0 x 10^4^ PFU/ml viral titer by formulation A of aCu fabric. **(G, H)** Deactivation of 3.0 x 10^4^ PFU/ml viral titer of bacteriophage on 2-year-old aCu-coated aluminum substrate over short duration. **(I, J)** Filter metal testing from collected water **(I)** and ICP-MS **(J)** after shedding/particulate testing. Fe indicates traces of stainless steel – stainless steel sample holders were used. Ca indicates environmental contamination/sample hygiene. Spike of copper at treated normal flow 2 is commensurate with other increases, indicating environmental sample contamination. **(K)** Results of ISO Test Method 18184:2019 antiviral textile test against feline calicivirus and influenza A (H3N2 & H1N1).

As an initial rapid efficacy pre-screen, we tested aCu against *Salmonella enterica*-specific bacteriophage Ø32 with exposure times of 5, 30, and 60 minutes. All active samples showed 100% viral kill rates after just 5 minutes; no plaques remained to be counted (**Figure 4A**). To ensure that these dramatic results were accurate, this test was duplicated on a different rayon/polyester (70/30% w/w) fabric blend where it yielded the same result (**Figure 4B**).

To determine how quickly aCu acts against microbes, we opted to further decrease the testing time increments to as short as 30 sec and test the kill rate upon initial contact.

Cellulose/polyester (45/55) samples were tested with two viral titers for the “ultra-rapid” efficacy test: ~3.0 x 10^4^ PFU/ml & ~3.0 x 10^5^ PFU/ml *Salmonella enterica*-specific bacteriophage Ø32. Viral challenges of *Salmonella* bacteriophage were administered to each of the samples. The surviving phages were measured at seven intervals (T0, T30s, T1, T2, T2.5, T3, T5 min). At T0, reductions of 60.6% (**Figures 4C, D**) and 60.3% (**Figures 4E, F**). By T30s, the viral titer was reduced by 86.6% and 88% (**Figures 4C, E**). T2, the samples had achieved 100% kill rates of both phage concentrations (**Figures 4C–F**). This compares very favorably to 70% ethanol, the disinfectant regarded as the standard, which achieved a 3 log_10_ reduction of SARS-CoV-2 after 30 seconds (Leslie et al., 2020), and a 2 log_10_ reduction of feline calicivirus after one minute (Malik et al., 2006).

Our subsequent test measured performance over an extended period of use to address the durability requirements established by the Environmental Protection Agency (EPA) for the registration of copper alloys as antimicrobial agents. Regulations stipulate that compounds must be able to continuously reduce a recurrent bacterial load (Blackburn, 2008), which would be expected in a hospital setting or on surfaces in public transportation.

Over 14 days, aCu’s sustained antimicrobial efficacy was tested according to the EPA protocol, which calls for repeated inoculations over the course of 21 hours (Blackburn, 2008). We lengthened the timeline to better gauge the longevity we could expect from copper-coated fabrics (**S2 Table**) and applied a very large viral load of 2·7x10^7^ PFU/ml to simulate high touch-traffic scenarios. The first extended exposure test was planned for 10 days anticipating some performance degradation. aCu-coated cellulose/polyester (45/55) fabric was treated with a daily titer of *Salmonella* bacteriophage Ø32 (2·7x10^7^ PFU/ml). After 10 days, antimicrobial capacity showed no degradation whatsoever. We opted to extend the test to 14 days. After 14 days the aCu-coated sample had maintained its antiviral capacity (**S2 Figure**). The material was as active against phages on day 14 as on day 1. The results of this test demonstrate the high efficacy of aCu even when subjected to the frequent and intense pathogen exposure that is a fixture of health care settings.

**Figure 5:**
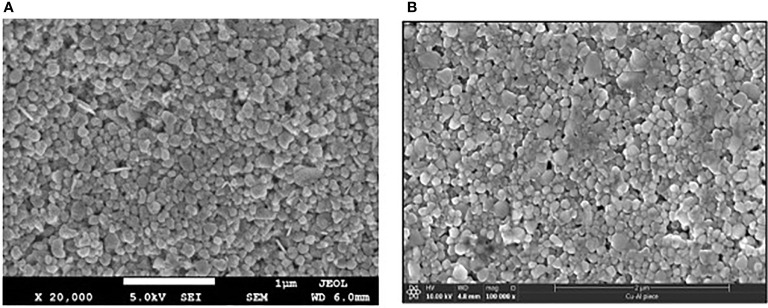
SEM image of two-year-old copper coating layer on Al substrate. **(A)** after initial coating in 2018; **(B)** 2 years later. Both show very similar agglomerated particle morphology, shape and size (images have been sized to reflect identical scale).

We wanted to understand what kind of durability and longevity we could expect from solid surfaces coated with a layer of aCu paint. A 2x2 cm, 3 mm thick piece of aluminum that had been fully coated with aCu paint in 2018 stored indoors for three years in ambient conditions with full exposure to seasonal temperature swings was used for the viral challenge test. By T15 s, 88% of the phages had been killed, by T30 s 89.3%, and by T60 s fully 100% of the phages had been killed, leaving no viral plaques to count (**Figures 4G, H** and **S5 Table**). SEM analysis revealed that the copper material had not changed its morphology over the two years, indicating excellent shelf-stability (**Figure 5**). From these data we learned that aCu maintains vigorous antimicrobial activity independent of its substrate; textiles and hard surfaces are both viable mediums.

Encouraged by aCu’s durability and fast action, we conducted efficacy testing based on ISO test #18184:2019 – Determination of Antiviral Activity of Textile Products. aCu was applied to cellulose/polyester fabric with additional CuCl_2_ as an activator. Samples were challenged with human influenza A (H1N1 & H3N2) and feline calicivirus VR-782. Viral suspensions were deposited onto the fabric samples and after two hours (shortest allowable with ISO) of incubation the aCu textile had reduced the concentrations of all three viruses by more than 99% (**Figure 4K** and **S6a, b Tables**). H1N1 was reduced by 99.93%, H3N2 by 99.77%, and feline calicivirus by 99.42%. It is encouraging to yield favorable results against both enveloped (influenza) and non-enveloped (calicivirus) viruses. This dramatic efficacy makes aCu a very promising candidate for the development of self-sanitizing PPE.

**Figure 6:**
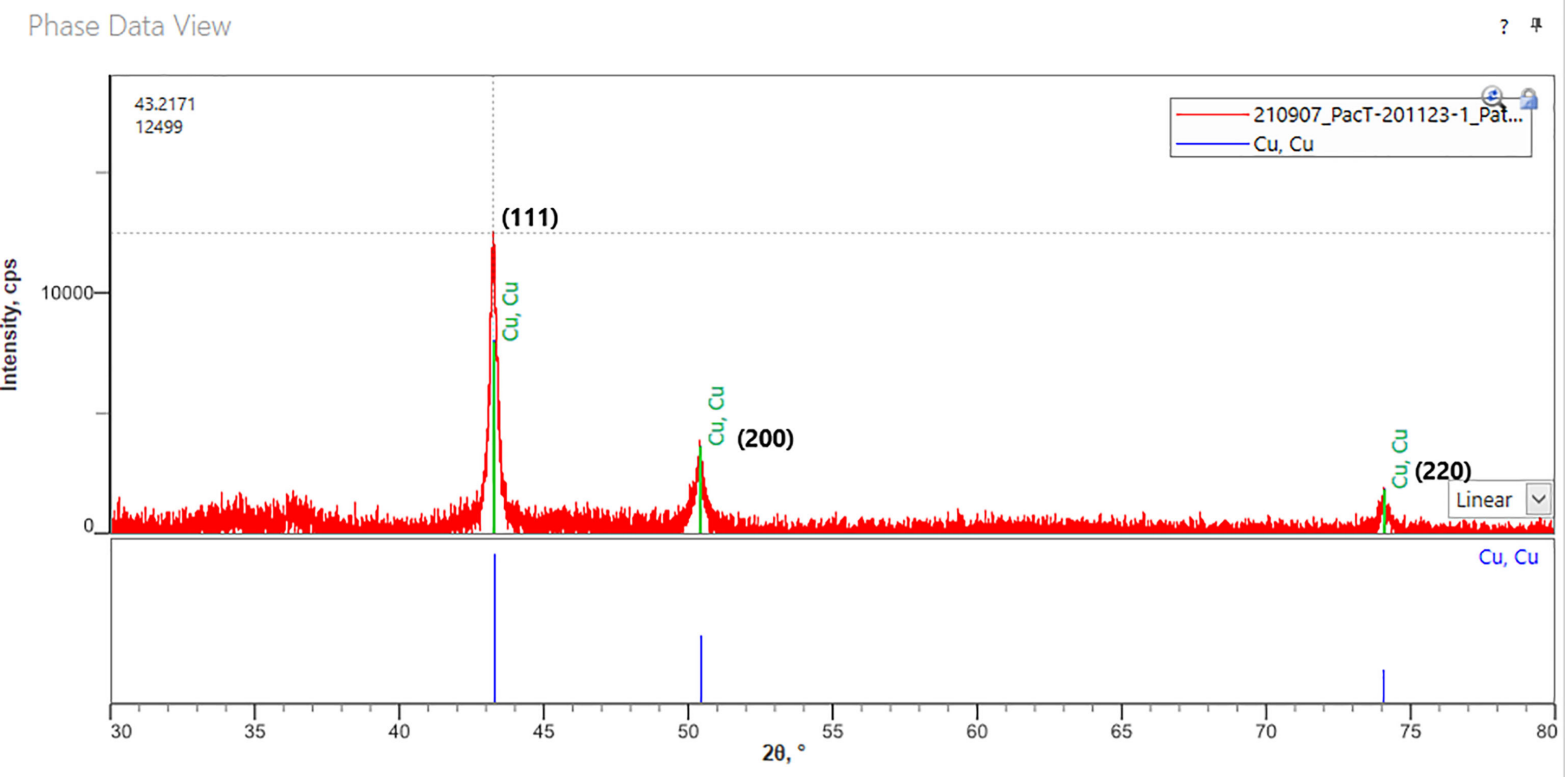
XRD taken of a typical fabric coated with ActiveCopper. The standard reference peaks for copper (111, 200, 220) are clearly noted on the XRD analysis, indicating minimal if any oxidation activity.

XRD analysis detected strong (111), (200) and (220) reflections of a copper metal phase, thus confirming the presence of metallic copper (**Figure 6**). The relatively large peak width indicates small particle size in the 100-200 nm range. Detection limit is about 1% and depends on crystallinity. There may be potential Cu_2_O in the 35° 2Theta area but is not raising sufficiently above the overall background noise to make a certain identification. Therefore, any oxide level is below 1%.

### Copper in Medicine and Mammalian Compatibility

To ensure that no harmful respiratory exposure occurs from using an aCu-coated insert in a face mask, shedding tests were conducted under simulated breathing conditions. aCu-coated fabric and blank controls were individually loaded into a sample holder and a flow of nitrogen was administered at rates commensurate with breathing. Two different flow rates were tested, 4.1 slpm and 20 slpm (standard liter per minute), to simulate normal breathing and more intense breathing during physical activity. A full battery of tests, including optical particle monitoring, condensation particle counting, filter trapping, and water trapping were employed to assess the exhausted N_2_ for shed particles (**Figures 4I, J**). It should be noted that these methods detect all potentially shed particles, including copper, fabric fibers, dust, or others. “Total particles” refers to all particles that lifted off the fabric under N_2_ flow. SEM/EDS analysis of the filter traps did not detect any copper (**S2, S4 Figures**). Metal levels in the water trapped from both treated and blank control materials were equivalent; no Cu was detected (**Figure 6I**). Optical particle counter tests found total particle counts to be <0.1 ng in each 15-minute test window (**S4 Figure**). These particles were organic in nature and looked like loose fabric material. Hypothetically, if all of the identified particles were copper, it would equate to a maximum shedding rate of 0·1 ng Cu per 15-minute period. This would translate to a maximal copper “dose” of approximately 2 ng/hour for a wearer. This would be significantly below OSHA’s maximum Permissible Exposure Limit (PEL) of 1 mg/m^3^ for copper dust in the workplace (National Institute for Occupational Safety and Health, (NIOSH), 2016). In addition to this flow-through testing, the EPA-standard GCMS testing indicated no off-gassing of amines, ketones, halogenated compounds or other aliphatic or aromatic compounds even when the samples were exposed to moisture and agitation (**S4 Table**). The simulated breathing and VOC data offers reassurance that aCu-coated fabrics are safe for frequent human use and proximity to the respiratory system. The moisture and agitation data provide further confirmation that even when exposed to moisture from respiration and sweat during long wear intervals, the masks do not present health risks.

Our results far exceed the benchmarks established by the EPA for speed of action as well as material longevity and durability. The favorable results against multiple virus configurations (enveloped and non-enveloped) suggest that aCu is broadly lethal against pathogens and is therefore unaffected by the possibility of future mutations. Enveloped viruses, like coronaviruses and influenzas, are generally regarded as less hardy than non-enveloped viruses (Kramer et al., 2006). aCu’s non-selectivity coupled with its rapid deactivation window suggests viability as an additive to healthcare tools like privacy curtains, door handles and guard rails.

As with any material that contacts skin or the respiratory system, safety is of utmost concern. A protective item must not introduce new health threats while protecting the user. To this end, it’s pertinent to highlight copper’s long and safe history in medical and industrial materials (Longano et al., 2011; Hordyjewska et al., 2014). Ancient civilizations recorded medical copper use as early as 2200 B.C., and a more scientific understanding was established when a doctor realized that copper miners and smelters enjoyed distinct immunity from Paris’s 1832 cholera outbreak (Grass et al., 2011). Reliance on copper continued to grow until it was displaced by penicillin in 1932. More recently, copper has been incorporated into medical devices (United States Food and Drug Administration, 2020), dental cement (Glassman and Miller, 1984; Mahler, 1997), and dilute CuCl_2_ IV solutions to treat copper deficiencies in patients (Griffith et al., 2009). Copper’s bioactivity is relevant in both the synthesis and maintenance of skin proteins; it is used in treating skin conditions (Borkow, 2014). Extended dermal exposure to copper has been shown to be well-tolerated (Borkow, 2013).

Copper plays an essential role in human health. It is involved in angiogenesis (Gérard et al., 2010), transcription factor regulation, bone development, and various other physiological processes (Uauy et al., 1998). Deficiencies in bodily copper can cause anemia, neutropenia, and neurological disorders (Uauy et al., 1998; Desai and Kaler, 2008). Because it is so well integrated into human biology, robust mechanisms exist to regulate copper levels in the body (Turnlund, 1998). Homeostatic maintenance of copper occurs *via* intestinal absorption and release is mediated by the liver into bile (Prohaska, 2008). Small stores of copper are kept in the body, approximately 0.5 mg per pound of body weight in the average adult (Erdman et al., 2012).

## Discussion

When the EPA recognized the antimicrobial efficacy of copper alloys in February, 2008, copper’s biocompatibility contributed to their determination that “these products pose no risks to public health; copper products have been in use for centuries, and we know of no harm from such use” (Swindell, 2008). EPA recognition allows copper manufacturers to claim that approved copper products “kill 99.9% of bacteria within two hours” (Swindell, 2008). The two-hour guarantee of bacterial deactivation is accompanied by an EPA mandate to maintain rigorous disinfecting regimens because copper that works on this timescale could still transmit pathogens. Over 500 copper alloys are registered with the EPA and deemed to have no “unreasonable adverse effects” under the U.S. Federal Insecticide, Fungicide, and Rodenticide Act (Swindell, 2008).

Because our testing indicated that aCu has the potential to function as personal protective equipment in a medical setting, it is necessary to consider the precedent set by other medical copper products prior to the development of aCu for safety and indicators of need within the field. We considered the guidance from the Food and Drug Administration (FDA). The FDA has listed fine copper powder (95% w/w Cu) as exempt from certification, deeming it safe for use as a color additive in cosmetics. This qualification was extended to products intended for use specifically in the eye area (U.S. FDA, 2017). The FDA’s relaxed position on copper provided further reassurance that our material would create no health issues for the people using it. FDA approval has been sought and granted for other medical copper products, establishing a precedent for copper’s enduring modern utility. FDA approval was granted for a breathable face mask in 2016 (U.S. FDA., 2009 *)* that protects against bacteria and influenza viruses. The mask material was proven to kill 99% of tested bacteria after one hour of contact and inactivate 99.9% of test influenza viruses after five minutes of contact (U.S. FDA., 2009).

The antimicrobial technology at play applies fine zeolite infused with Ag^+^ and Cu^+^ ions to the non-woven polyester fibers of a mask. Their application method eliminated any off-gassing or leaching of the antimicrobial agent, which is what we have accomplished with aCu (**S4 Table**). To securely fix aCu paint to the intended material, we have adopted the practice of gluing (bonding) aCu to the core of the fabrics. The resulting aCu-coated fabric retains all of its original flexibility; it does not become rougher or stiffer following the addition of aCu. The play in the fabric is important, because it helps ensure that the fabric will be comfortable for extended periods of wear. aCu’s protective lipid-type layer both protects and stabilizes the high surface area of aCu, further enabling strong cohesion of the micron-size agglomerates (**Figure 1**). Our highly active copper material is differentiated from related precursors by its ability to kill viruses and bacteria in one minute or less.

Using a mask filter insert that is rapidly antimicrobial comes with a novel benefit: it is self-sterilizing. The washable outer mask can be laundered as many times as necessary, while the insert remains clean *via* self-sterilization. Our long term 14-day test showed that after two weeks, the aCu fabric was still as effective against microbes as it had been on the first day. Part of our rationale for conducting long term tests was to determine if aCu-treated PPE could be reusable. These results tell us that a mask insert could be reused for up to two weeks, offering a dramatic improvement over single-use PPE and its environmental burden.

We have speculated on the mechanisms responsible for aCu’s pathogen-agnostic eradication of microbes and have been informed extensively by the work of other researchers. Research on inorganic aerogels, xerogels and the like has shown that they exhibit high surface areas due to their fine pore structure; they are strongly attracted to one another and other surfaces *via* hydrophobic interactions or hydrophilic hydrogen bonding and chain entanglement (Liu et al., 2012). This can be enhanced by placing long-chain organic surfactants at their surfaces, forming a reverse micelle-type structure. We did exactly that with our aCu and designed a surfactant monolayer that maximizes attraction and mimics a lipid cell membrane. A virus’s small size (20-300 nm) combined with its commensurate surface energy to aCu results in mutual attraction. When they stick together, the protective lipid envelope and the protein capsid of the virus is readily attacked by aCu. The copper oxidizes first to Cu^+1^ and then more rapidly to Cu^+2^ with the formation of very aggressive OH* radicals in the presence of moisture and air as well as fatty acid radicals from the oxidation of the protective lipid layer:


$$2\;aCu^{0}+\;2\;H_{2}O\;+\;O_{2}\to 2\;CuOH\;+\;2\;HO*$$

$$2\;aCu^{0}+\;2\;FA-COOH\;+\;O_{2}\to 2\;CuOH\;+\;2\;FA-COO*$$

This process causes the lipid layer to disintegrate and the protein capsid to break open (Abad et al., 1994; Warnes and Keevil, 2013). The destruction of the capsid was identified by Dr. Sarah Warnes, et al. in 2015 using transmission electron microscopy (TEM) (Warnes et al., 2015). Viral replication is halted as Cu^2+^ ions, stabilized by their amine surfactants, cleave and cross-link RNA/DNA through their affinity for nucleotides (Ishida, 2018). This mechanism is analogous to the DNA binding exhibited by anti-tumor *cis*-platinum compounds (Roberts and Pascoe, 1972). Both reactive oxygen species and ionic interactions have been shown to facilitate viral deactivation. Due to the unprecedented speed and activity of aCu, more testing is necessary to elucidate the exact order of operations.

Action against bacteria begins as their unique cell wall degrades (Peters et al., 2018). Copper ions inhibit the transpeptidases responsible for cross-linking the peptidoglycan wall, compromising its structural integrity. Bacteriolysis is furthered by copper’s interruption of the necessary balance between peptidoglycan-synthesizing enzymes and autolysin (Peters et al., 2018). The destruction of such a fundamental piece of the bacterial framework in both Gram negative and positive strains indicates that aCu’s efficacy would not be hindered by bacterial mutation. Additionally, antimicrobial copper experiments have been successfully conducted against a number of antibiotic-resistant strains. Reactive oxygen species generated by Cu^2+^’s interactions with the cell wall and subsequent permeation into the membrane may harm bacteria *via* cytoplasmic toxicity (Solioz et al., 2010). aCu does not act as a mutagen, as antibiotics can (Long et al., 2016), indicating that it is not liable to produce mutant bacterial strains and contribute to antibiotic resistance (Santo et al., 2011).

Around the world, people need to return to life, work, and family while protecting their health. A growing number of people who experienced flu-like Covid-19 and were believed to have recovered are reporting persistent fatigue, gastrointestinal, and cognitive issues that outlast their original symptoms (Nabavi, 2020). In some cases, these long-term impacts manifest as cardiac dysfunction (Mahase, 2020). Trailing symptoms are not unprecedented with coronaviruses; a study conducted in 2009 on survivors of SARS determined that 40% of respondents struggled with chronic fatigue (Lam et al., 2009). In addition to the detrimental follow-on effects of Covid-19, it is becoming apparent that reinfection may be possible (Tillett et al., 2020). The uncertainty that surrounds the long-term consequences of contracting Covid-19 puts additional pressure on people to mitigate their risk, no matter their age.

We have just witnessed the most rapid development of a vaccine in history (Soleimanpour and Yaghoubi, 2021). Major logistical hurdles remain before the desired rate of distribution can be reached. At this moment, public distrust in medicine and governing authorities translates to considerable vaccine hesitancy in a multitude of countries (Hornsey et al., 2020; Thunstrom et al., 2020; Coustasse et al., 2021). This apprehension will likely hinder the compliance necessary to achieve broad inoculation and subsequent immunity (Neumann-Böhme et al., 2020). The lightning-fast arrival of multiple effective vaccines has been an unequivocal triumph. However, a long-lasting protective mask offers individuals an immediate and reliable defense against a wide range of pathogens, including an annual flu that hits our society every year. The use of a protective mask is both reversible and personally administered; a vaccine is not.

Remedies targeted specifically at SARS-CoV-2 are in dire need, but in the long term we need broadly transportable, non-selective protection against pathogens. Although the medical realm has been dominated by Covid-19 for all of recent memory, everyone is aware that unrelated pathogens and mutant strains still pose an enduring threat. In real time we have watched SARS-CoV-2 jump from humans to large populations of farmed mink in Denmark and now we are seeing a second episode of zoonosis as farm workers become sick with the mink-mutated strain (Enserink, 2020). Genomic sequencing confirmed that this strain was transmitted from humans to animals and back once more. When given the opportunity to move through a dense concentration of hosts, such as a farm, viruses can mutate into more virulent forms much more quickly than they would in the wild (Boots et al., 2004). A reassortant H1N1 influenza virus with pandemic potential has already been identified in China (Sun et al., 2020), and we can continue to count on the appearance of seasonal illnesses. Due to the lag time in development, a situation is conceivable where the distributed vaccine would miss the currently circulating coronavirus mutant. Finally, single-use PPE creates an enormous environmental impact that could be significantly mitigated by reusable options (Karim et al., 2020). We posit that masks and other protective pieces that self-sterilize to destroy 100% of pathogens could aid in an expedited return to uninterrupted life.

## Data Availability Statement

The original contributions presented in the study are included in the article/**Supplementary Material**. Further inquiries can be directed to the corresponding authors.

## Author Contributions

AZ originally conceived of and developed ActiveCopper, and manages all projects related its use. MI and HK conducted foundational antimicrobial research RB wrote the paper, designed figures, and compiled data RR, AV, KN, NN, and HZ produced and managed testing materials, formulated the copper paste, troubleshot technical materials issues NA supervised the project and handled administration and funding acquisition RS handles the commercial manufacturing and scale up of the materials, developed methodologies, and supervised the projects. All authors contributed to the article and approved the submitted version.

## Funding

This study was independently funded by Kuprion®, Inc. The funder was not involved in the study design, collection, analysis, interpretation of data, the writing of this article or the decision to submit it for publication. All authors declare no other competing interests.

## Conflict of Interest

Authors AAZ, MI, RLB, RR, AV, KKN, NTN, HTZ, NA, and RMS are employed by Kuprion^®^, Inc. Authors MI and HK are employed by Integrated Pharma Services. This study was funded by Kuprion^®^, Inc. All authors declare no other competing interests.

## Publisher’s Note

All claims expressed in this article are solely those of the authors and do not necessarily represent those of their affiliated organizations, or those of the publisher, the editors and the reviewers. Any product that may be evaluated in this article, or claim that may be made by its manufacturer, is not guaranteed or endorsed by the publisher.
