# Supplementary material for: Rapidly Self-Sterilizing PPE Capable of Destroying 100% of Microbes in 30-60 Seconds
Source: Front Cell Infect Microbiol. 2021 Dec 15;11:752899. doi: 10.3389/fcimb.2021.752899 (PMC8715083; doi:10.3389/fcimb.2021.752899)
Supplement: Supplementary file 1 [file DataSheet_1.pdf]

Supporting information for: Rapidly Self-Sterilizing PPE Capable of Destroying 100% of Microbes in 30-60 Seconds.

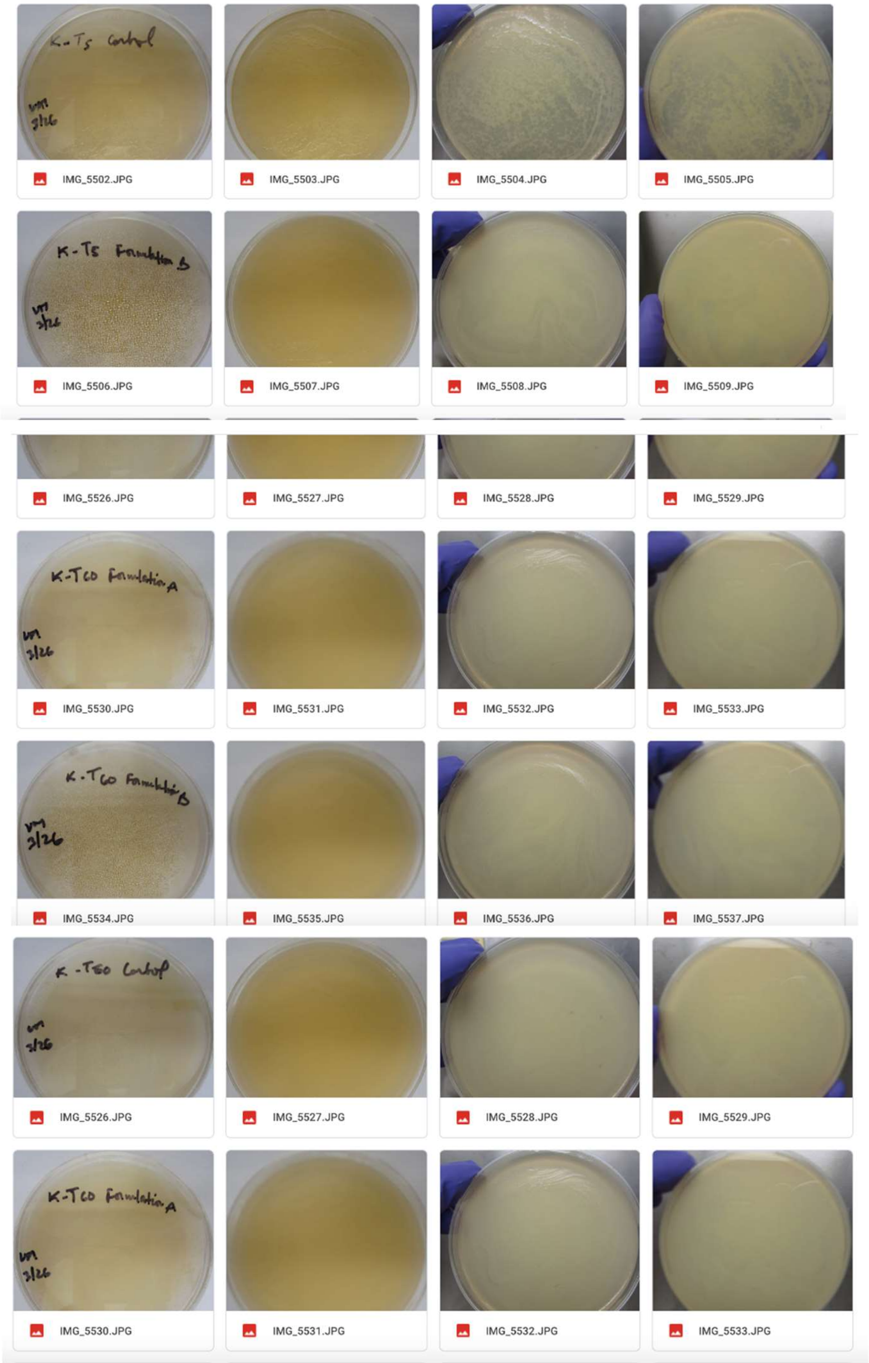

S1 Fig. Images from initial test at T5 & T60 comparing bacteriophage growth between active fabrics and control. A Control & sample B at T5, B Formulation A & B at T60, C Control & Formulation A at T60.

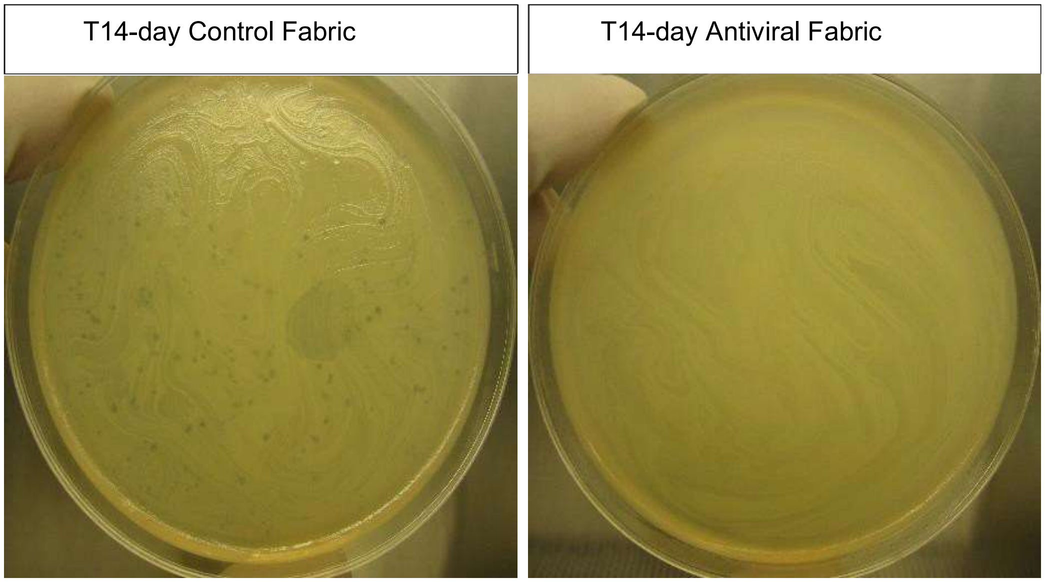

S2 Fig. Comparison between control and active aCu fabric after 14-day test.
Comparison shows prolific viral plaques on control fabric and no viral plaques on antiviral fabric, indicating 100% kill rate against 10^7 pfu/ml challenges.

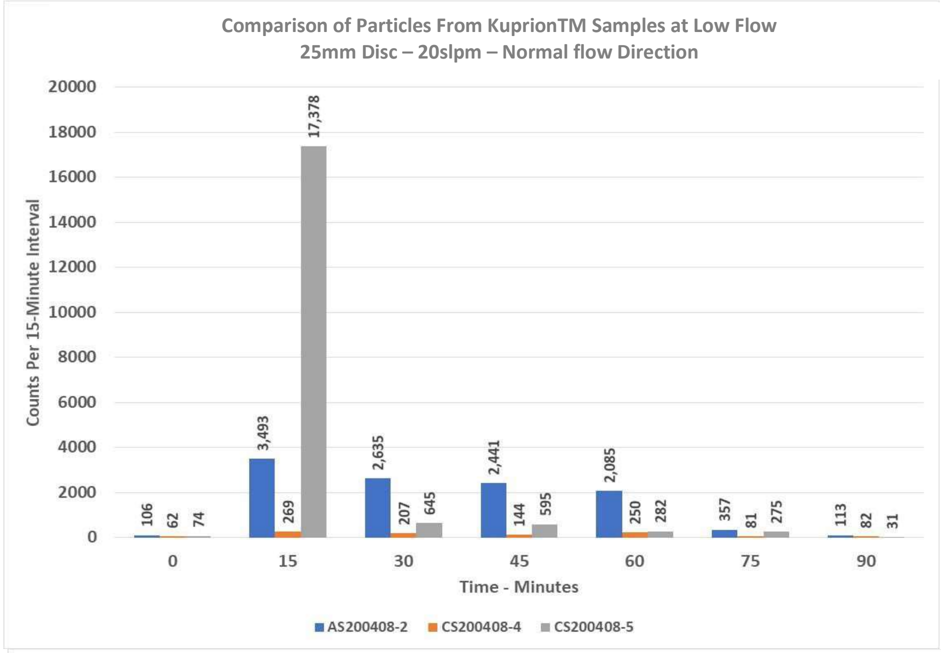

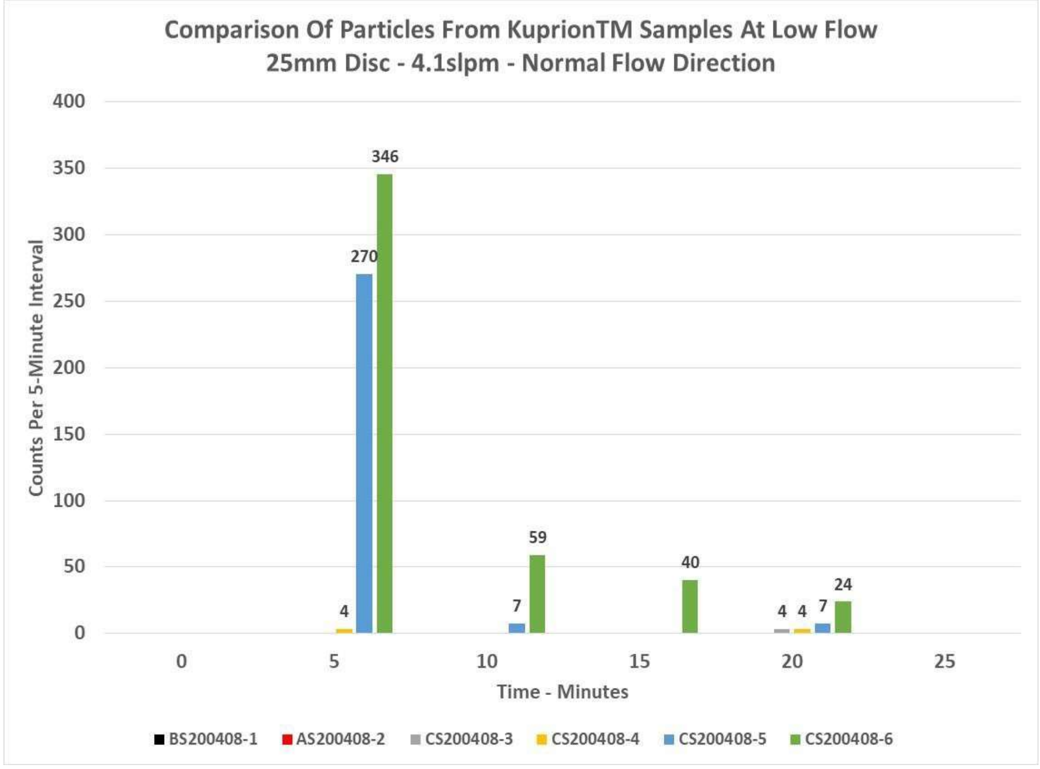

S3 Fig. Comparison of particles from high/low flow rate during breathing simulation test. CS200408-5 at 15 minutes may be an erroneous read.

EDS Analysis

MilliporeSigma Imaging Lab

KUP-T-N-1 Treated Fabric, Normal Flow, Iteration #1

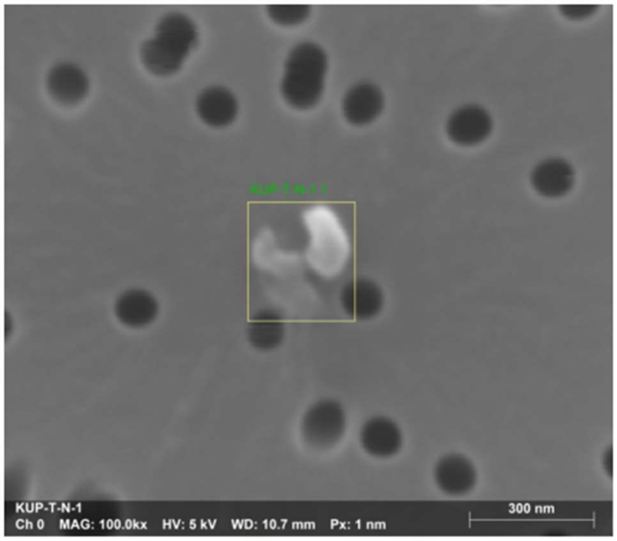

Name	Date	Time	HV [kV]	Mag	WD [mm]
KUP-T-N-1	4/28/2020	10:52:12 AM	5.0 keV	100000x	10.7 mm

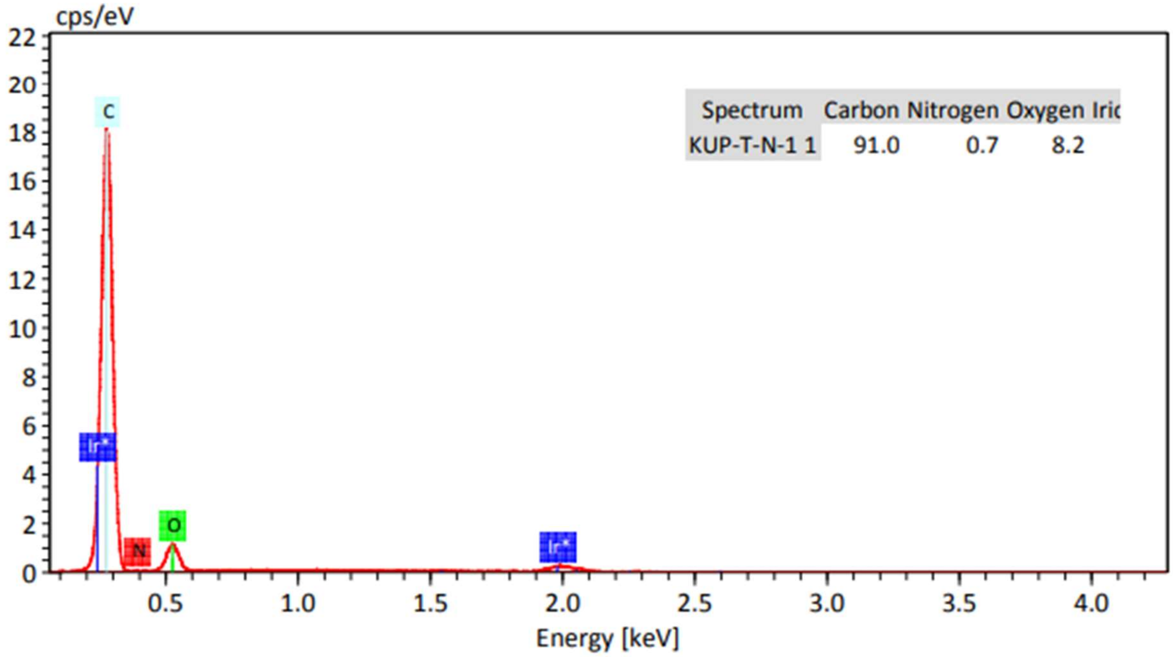

S4 Fig. SEM-EDS analysis filter trap after flow of nitrogen through treated fabric. Iridium metallization of the samples was used to reduce charging.

Sample ID	Description	Ship Date	Spray Size	Sample Size	Qty.	Substrate	Spray Dist.	Binder (front)	Cu (front)	Cu (back)	Binder (front)	Binder (back)
BS200323-1	Blank Sample	3/23/2020	9" x 9"	2" x 2"	20	MicroTek (45/50)	N/A	none	none	none	none	none
CS200323-1	Cu Sample	3/23/2020	9" x 9"	2" x 2"	20	MicroTek (45/50)	~5	2x	3x	2x	none	none
CS200323-2	Cu Sample	3/23/2020	9" x 9"	2" x 2"	20	MicroTek (45/50)	~5	2x	3x	2x	none	none
CS200325-8	Cu Sample	3/25/2020	9" x 9"	2" x 2"	20	MicroTek (45/50)	~5	2x	**5x**	2x	none	none
CS200325-9	Cu Sample	3/25/2020	9" x 9"	2" x 2"	20	MicroTek (45/50)	~5	2x	3x	2x	none	none
BS200401-1	Blank Sample	4/1/2020	9" x 9"	2" x 2"	20	U-T Rayon/Poly (70/30)(roll)	N/A	none	none	none	none	none
CS200401-1	Cu Sample	4/1/2020	9" x 9"	2" x 2"	20	U-T Rayon/Poly (70/30)(roll)	~5	2x	3x	none	1x	none
CS200401-2	Cu Sample	4/1/2020	9" x 9"	2" x 2"	20	U-T Rayon/Poly (70/30)(roll)	~5	2x	1x	none	1x	none
BS200408-1	Blank Sample	4/8/2020	12" x 12"	12" x 12"	1	MicroTek (45/50)	N/A	none	none	none	none	none
AS200408-2	Adhesive Sample	4/8/2020	12" x 12"	12" x 12"	1	MicroTek (45/50)	3"	2x	none	none	none	none
CS200408-3	Cu Sample	4/8/2020	12" x 12"	12" x 12"	1	MicroTek (45/50)	3"	2x	3x	2x	none	none
CS200408-4	Cu Sample	4/8/2020	12" x 12"	12" x 12"	1	MicroTek (45/50)	3"	2x	5x	2x	none	none
CS200408-5	Cu Sample	4/8/2020	12" x 12"	12" x 12"	1	MicroTek (45/50)	3"	2x	3x	2x	1x	none
CS200408-6	Cu Sample	4/8/2020	12" x 12"	12" x 12"	1	MicroTek (45/50)	N/A	none	3x	2x	none	none
BS200416-1	Blank Sample	4/16/2020	12" x 12"	2" x 2"	25	MicroTek (45/50)	N/A	none	none	none	none	none
BS200416-2	Cu Sample	4/16/2020	12" x 12"	2" x 2"	25	MicroTek (45/50)	3"	2x	3x	2x	none	none
CS200416-3	Cu Sample	4/16/2020	12" x 12"	2" x 2"	25	MicroTek (45/50)	3"	2x	3x	2x	none	none
BS200416-1	Blank Sample	4/16/2020	N/A	2" x 2"	25	MicroTek (45/50)	N/A	none	none	none	none	none
BS200427-1	Blank Sample	4/27/2020	10" x 11"	2" x 2"	25	U-T Rayon/Poly (70/30)(roll)		none	none	none	none	none
CS200427-2	Cu Sample	4/27/2020	10" x 11"	2" x 2"	20	U-T Rayon/Poly (70/30)(roll)		2x	2x	none	1x	none

S1 Table. Fabric samples used for testing.

This comprehensive list of samples includes date of manufacture, destination, size, material, coating, and labeling convention.

Smpl #	Inoculation Date										Titer check	Incubation time
	1st	2nd	3rd	4th	5th	6th	7th	8th	9th	10th		
1	4/21(Tue)										4/23(Thu)	1-day
2	4/21(Tue)	4/22(Wed)									4/24(Fri)	2-day
3	4/21(Tue)	4/22(Wed)	4/23(Thu)								4/27(Mon)	3-day
4	4/21(Tue)	4/22(Wed)	4/23(Thu)	4/24 (Fri)							4/28(Tue)	6-day
5	4/21(Tue)	4/22(Wed)	4/23(Thu)	4/24 (Fri)	4/27(Mon)						4/29(Wed)	7-day
6	4/21(Tue)	4/22(Wed)	4/23(Thu)	4/24 (Fri)	4/27(Mon)	4/28(Tue)					4/30(Thu)	8-day
7	4/21(Tue)	4/22(Wed)	4/23(Thu)	4/24 (Fri)	4/27(Mon)	4/28(Tue)	4/29(Wed)				5/1(Fri)	9-day
8	4/21(Tue)	4/22(Wed)	4/23(Thu)	4/24 (Fri)	4/27(Mon)	4/28(Tue)	4/29(Wed)	4/30(Thu)			5/4(Mon)	10-day
9	4/21(Tue)	4/22(Wed)	4/23(Thu)	4/24 (Fri)	4/27(Mon)	4/28(Tue)	4/29(Wed)	4/30(Thu)	5/1(Fri)		5/5(Tue)	13-day
10	4/21(Tue)	4/22(Wed)	4/23(Thu)	4/24 (Fri)	4/27(Mon)	4/28(Tue)	4/29(Wed)	4/30(Thu)	5/1(Fri)	5/4(Mon)	5/6(Wed)	14-day

S2 Table. 14-day inoculation schedule for EPA long-term efficacy test. Fabric samples were inoculated and removed according to the matrix above.

Time Point	Formulation C (PFU/ml)	Formulation D (PFU/ml)	Formulation C (PFU/ml)	Formulation D (PFU/ml)
	Ø32 (2.7×10^5 PFU/ml)		Ø32 (2.7×10^4 PFU/ml)	
**T0**	2.55×10^5	2.08×10^5	1.64×10^4	1.85×10^4
**T30s**	6.90×10^4	2.10×10^4	4.00×10^3	3.60×10^3
**T1**	5.50×10^4	5.00×10^3	7.00×10^2	8.00×10^2
**T2**	1.00×10^3	0	0	0
**T2.5**	7.00×10^2	0	0	0
**T3**	0	0	0	0
**T5**	0	0	0	0
**Control**	0	0	0	0

S3 Table. Results from ultra-rapid EPA efficacy test. Formulations C and D were tested from times T0-T2.5, and the remaining PFUs are tabulated.

Parameters	MDL	PQL	Method Blank Conc.	Lab Qualifier	
Dichlorodifluoromethane	0.061	0.50	ND		
Chloromethane	0.091	0.50	ND		
Vinyl Chloride	0.10	0.50	ND		
Bromomethane	0.13	0.50	ND		
Chloroethane	0.15	0.50	ND		
Trichlorofluoromethane	0.10	0.50	ND		
1,1-Dichloroethene	0.10	0.50	ND		
Freon 113	0.094	0.50	ND		
Methylene Chloride	0.36	0.50	ND		
trans-1,2-Dichloroethene	0.10	0.50	ND		
MTBE	0.12	0.50	ND		
TBA	0.58	2.5	ND		
Diisopropyl ether	0.11	0.50	ND		
1,1-Dichloroethane	0.11	0.50	ND		
Ethyl tert-Butyl ether	0.11	0.50	ND		
cis-1,2-Dichloroethene	0.11	0.50	ND		
2,2-Dichloropropane	0.096	0.50	ND		
Bromochloromethane	0.12	0.50	ND		
Chloroform	0.12	0.50	ND		
Carbon Tetrachloride	0.10	0.50	ND		
1,1,1-Trichloroethane	0.10	0.50	ND		
1,1-Dichloropropene	0.099	0.50	ND		
Benzene	0.11	0.50	ND		
TAME	0.11	0.50	ND		
1,2-Dichloroethane	0.12	0.50	ND		
Trichloroethylene	0.090	0.50	ND		
Dibromomethane	0.092	0.50	ND		
1,2-Dichloropropane	0.093	0.50	ND		
Bromodichloromethane	0.098	0.50	ND		
cis-1,3-Dichloropropene	0.080	0.50	ND		
Toluene	0.091	0.50	ND		
Tetrachloroethylene	0.083	0.50	ND		
trans-1,3-Dichloropropene	0.082	0.50	ND		
1,1,2-Trichloroethane	0.092	0.50	ND		
Dibromochloromethane	0.094	0.50	ND		
1,3-Dichloropropane	0.092	0.50	ND		
1,2-Dibromoethane	0.090	0.50	ND		
Chlorobenzene	0.091	0.50	ND		
Ethylbenzene	0.083	0.50	ND		
1,1,1,2-Tetrachloroethane	0.097	0.50	ND		
m,p-Xylene	0.16	0.50	ND		
o-Xylene	0.086	0.50	ND		
Styrene	0.082	0.50	ND		
Bromoform	0.084	0.50	ND		
Isopropyl Benzene	0.081	0.50	ND		
Parameters	MDL	PQL	Method Blank Conc.	Lab Qualifier	
n-Propylbenzene	0.078	0.50	ND		
Bromobenzene	0.088	0.50	ND		
1,1,2,2-Tetrachloroethane	0.096	0.50	ND		
2-Chlorotoluene	0.088	0.50	ND		
1,3,5-Trimethylbenzene	0.079	0.50	ND		
1,2,3-Trichloropropane	0.095	0.50	ND		
4-Chlorotoluene	0.082	0.50	ND		
tert-Butylbenzene	0.081	0.50	ND		
1,2,4-Trimethylbenzene	0.068	0.50	ND		
sec-Butyl Benzene	0.078	0.50	ND		
p-Isopropyltoluene	0.073	0.50	ND		
1,3-Dichlorobenzene	0.083	0.50	ND		
1,4-Dichlorobenzene	0.086	0.50	ND		
n-Butylbenzene	0.073	0.50	ND		
1,2-Dichlorobenzene	0.089	0.50	ND		
1,2-Dibromo-3-Chloropropane	0.092	0.50	ND		
Hexachlorobutadiene	0.068	0.50	ND		
1,2,4-Trichlorobenzene	0.074	0.50	ND		
Naphthalene	0.084	0.50	ND		
1,2,3-Trichlorobenzene	0.083	0.50	ND		
2-Butanone	0.11	0.50	ND		
4-Methyl-2-Pentanone	0.10	0.50	ND		
(S) Dibromofluoromethane			103		
(S) Toluene-d8			97.9		
(S) 4-Bromofluorobenzene			98.2		

S4 Table. Results from EPA Standard GCMS Analysis of agitated and tumbled aCu-coated fabric samples. ND indicates Not Detected, (*S*) indicates not applicable to the test administered.

Time Point (sec or min)	PFU/ml recovered
**T0**	1.1×10^4
**T15s**	3.0×10^3
**T30s**	1.4×10^3
**T1**	0
**T1.5**	0
**T2**	0
**T3**	0
**Control (PBS)**	0

S5 Table. aCu-coated aluminum piece exposure results. The sample was tested after inoculation according to the above table and the PFU/ml recovered from each inoculation is listed in the table.

Item	Antiviral Efficacy value (Mv)	Standard
Tested textile product	$3.0 > Mv \geq 2.0$	Good Effect
	$Mv \geq 3.0$	Excellent Effect

S6a, b Tables. Antiviral Performance Standard and results of ISO Antiviral Activity Test. All three viruses, feline calicivirus and influenza A H3N2 & H1N1, were all significantly diminished by contact with the aCu fabric.

**Virus**	**Antiviral Value**	**Antiviral Property**	**Percent Reduction**
Feline Calcivirus	2.48 and 2.08	Yes	99.42%
Influenza A (H3N2)	2.63	Yes	99.77%
Influenza A (H1N1)	3.0 and 3.34	Yes	99.93%
